# A least-squares-fitting procedure for an efficient preclinical ranking of passive transport across the blood–brain barrier endothelium

**DOI:** 10.1007/s10822-023-00525-1

**Published:** 2023-08-12

**Authors:** Christian Jorgensen, Evan P. Troendle, Jakob P. Ulmschneider, Peter C. Searson, Martin B. Ulmschneider

**Affiliations:** 1 Institute for NanoBioTechnology, Johns Hopkins University, Baltimore, MD USA; 2 Department of Chemistry, King’s College, London, UK; 3 Department of Materials Science and Engineering, Johns Hopkins University, Baltimore, MD USA; 4 School of Physics and Astronomy, Shanghai Jiao Tong University, Shanghai, China; 5 Department of Chemistry, Aarhus University, Langelandsgade 140, 8000 Aarhus C, Denmark

**Keywords:** Blood–brain barrier, Brain permeability, Molecular dynamics, Kinetics, Transport properties, CNS penetration

## Abstract

The treatment of various disorders of the central nervous system (CNS) is often impeded by the limited brain exposure of drugs, which is regulated by the human blood–brain barrier (BBB). The screening of lead compounds for CNS penetration is challenging due to the biochemical complexity of the BBB, while experimental determination of permeability is not feasible for all types of compounds. Here we present a novel method for rapid preclinical screening of libraries of compounds by utilizing advancements in computing hardware, with its foundation in transition-based counting of the flux. This method has been experimentally validated for in vitro permeabilities and provides atomic-level insights into transport mechanisms. Our approach only requires a single high-temperature simulation to rank a compound relative to a library, with a typical simulation time converging within 24 to 72 h. The method offers unbiased thermodynamic and kinetic information to interpret the passive transport of small-molecule drugs across the BBB.

**Graphical abstract:**

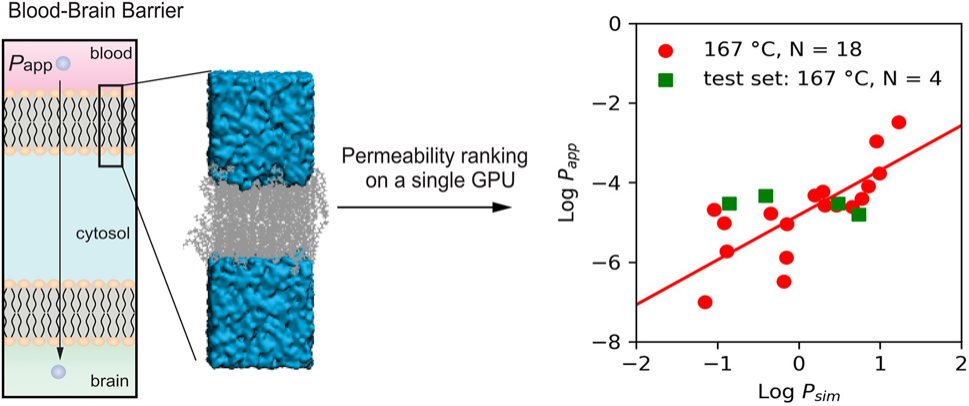

**Supplementary Information:**

The online version contains supplementary material available at 10.1007/s10822-023-00525-1.

## Introduction

**Fig. 1:**
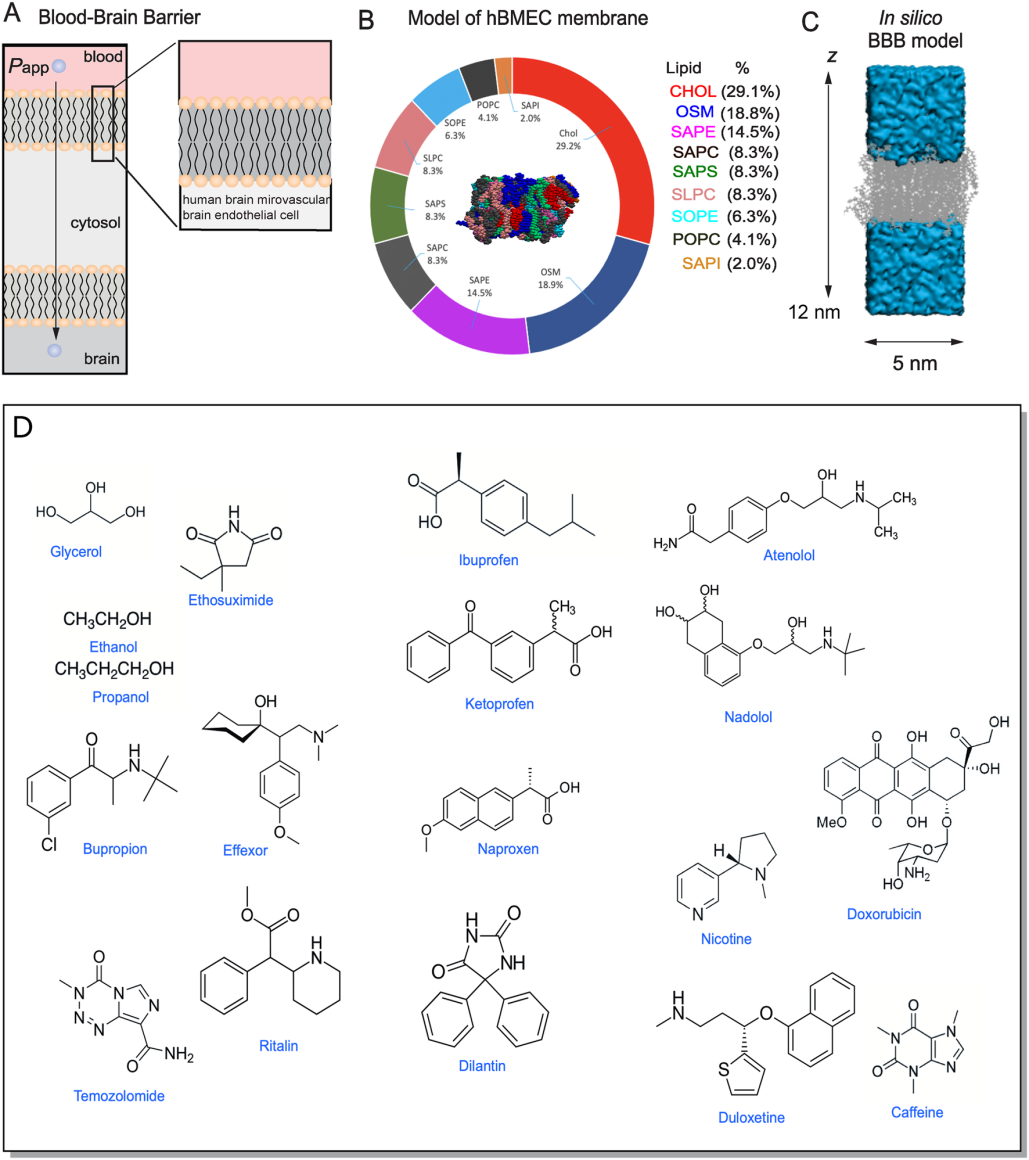
Construct of our in silico model of the blood–brain barrier for MD simulation of molecular transport across the BBB. **A** A representation of the human brain microvascular endothelial cell (hBMEC) membrane that forms a key element of the blood–brain barrier (BBB). **B** Composition of the in silico BBB apical hBMEC membrane model (N = 96 lipid molecules). **C** An in silico representation of the apical hBMEC membrane, showing dimensions and a drug crossing event. The system used has a system with a larger z-dimension (12 nm at equilibration), which is different (60% larger in z dimension than x–y dimensions) to our previous systems [26]. (D) Library of CNS compounds spanning a representative set of permeabilities (10^–7^ cm/s to 10^–3^ cm/s)

The treatment options for central nervous system (CNS) disorders are hindered by the blood–brain barrier (BBB), which is a highly selective barrier that regulates and restricts the transport of molecules from circulation into the CNS [1, 2]. The BBB is a complex physiological structure that separates the CNS from the bloodstream and is composed of a specialized layer of endothelial cells that line the blood vessels in the brain and spinal cord. The tight junctions between human brain microvascular endothelial cells (hBMECs; see Fig. 1) effectively prevent the paracellular transport of molecules and restricts transcellular transport into the brain to molecules with compatible chemical properties [3]. Therefore the primary mode for drugs to enter the brain is by passive diffusion across the luminal and abluminal hBMEC membranes. Furthermore, successful drugs must also not be substrates of BBB efflux pumps or need to diffuse sufficiently fast to overcome the action of efflux pumps. otherwise the effective concentration is significantly reduced. This means that the majority of drugs do not cross the BBB, making it difficult to deliver drugs to the CNS to treat neurodegenerative and psychiatric disorders.

The BBB plays a key physiological role in the development and progression of several neurodegenerative [4], including Alzheimer's disease [5], Parkinson's disease [6], multiple sclerosis and brain tumors [7]. In these and other CNS conditions, the BBB can become "leaky" which can lead to the accumulation of harmful molecules in the brain and contribute to the progression of these disorders. Additionally, molecular transport across BBB may also play a role in the development of psychiatric disorders [8] such as anxiety and depression [9], as well as schizophrenia [9] as the BBB also regulates the distribution of neurotransmitters and neurotrophic factors, which are essential for the proper functioning of the brain.

Therefore, the determination of the brain exposure of drugs is a challenging task that represents a major obstacle to the development of drugs for the treatment of CNS disorders. Despite the fact that there are approximately 1700 FDA-approved drugs, the brain exposure of only around 200 of these drugs is known [10, 11]. This lack of information about brain exposure makes it difficult to repurpose existing drugs for use in the treatment of CNS disorders. This highlights the need for new technologies that can identify therapeutics with the potential to cross the BBB early on in the drug development process [12, 13].

One of the more common screening models used for this purpose is Lipinski's rule of five [3], which is based on the idea that drugs with certain chemical properties, such as a low molecular weight and a neutral charge, are more likely to cross the BBB. Another methodology that is used is the QikProp program of Schrödinger, which uses the method of Duffy and Jorgensen [14]. This program uses a variety of cheminformatic methods to predict the properties of a drug, including its potential to cross the BBB. Generally, in silico models can be useful for identifying promising lead compounds early on in the drug development process, but existing solutions are limited in their ability to deliver unbiased atomic-detail insights into the physical process of BBB penetration. Atomistic insights are important to elucidate the underlying mechanism behind black-box or machine learning (ML) model predictions, which can only be obtained with MD simulations. As an example, two molecules can have their permeability predicted coarsely with ML models, but we can only rationalize the difference by observing the simulation trajectories.

Experimentally, the customary method for determining brain exposure of pharmaceutical compounds is the transwell assay, which is a widely used in vitro method [15]. In these assays, a BBB-mimetic confluent monolayer is used to mimic the tight junctions of the blood–brain barrier and the apparent permeability (*P*_app_) of the drug is determined by measuring the amount of the molecule that crosses the monolayer [16, 17]. Ideally, the monolayer used in this assay is composed of hBMECs (i.e., primary cells which would form the BBB in vivo), but a variety of primary cell types have been utilized for transwell assays, including confluent monolayers of Madin–Darby Canine Kidney (MDCK) or Caco-2 cells [16, 17], or more recently BMECs derived from induced pluripotent stem cells [18]. These experiments provide a means to evaluate the rate of transport of drugs across the BBB from estimating the content from the sacrificed rat brain. The data obtained from these experiments may then be compared to in silico simulations of transcellular transport to gain insights into the mechanisms of transport of drugs across the BBB [19–23].

Molecular dynamics (MD) simulations are a powerful computational tool that can be used to investigate the free energy of drug permeation across the transcellular pathway of the blood–brain barrier (BBB). MD simulations have been used to study a wide range of membrane types [19–23], including plasma and mammalian membrane models. There have been few studies that have attempted to simulate the endothelial membranes using accurate compositions [24–26], and some of these studies have revealed the computational challenges in converging permeability estimates.

To overcome sampling limitations in MD simulations for cellular translocation, various enhanced sampling techniques have been developed [27, 28]. However, these methods do not directly provide transport rates without a reweighting of the resulting ensemble or inferences through an inhomogeneous-solubility diffusion (ISD) framework [19]. Unbiased all-atom MD simulations provide detailed insights into the molecular mechanisms of transport across the BBB without the need for a priori knowledge of the permeation pathway or constrained coordinate systems. Kinetics can be calculated from the transition-based counting (TBC) approach [24, 29], but this still requires simulation times in the tens to hundreds of microseconds per drug partition to achieve converged estimates at 37 °C. This is currently beyond the limit of realistic sampling for routine MD simulations. Other methodologies for permeability have only been parametrized for the fast regime (permeability, *P* > 10^–5^ cm/s) [23].

In previous research, we have shown that high-temperature simulations can be used to estimate permeabilities at body temperature from direct Arrhenius fits [26] or by fitting the rates based on a modified form of Kramer's theory of reaction rate [24]. Although these simulations are run at much higher than physiological temperature, we have demonstrated that membranes do not disintegrate, nor do they significantly differ, even at extremely elevated simulated temperatures of 500 K (~ 226.85 °C). Furthermore, it has well-established in the MD simulation literature that the thermostat does not permit a vapour transition, and the membrane stability at high temperatures has also been experimentally validated [30, 31]. Both of these procedures have limitations, as the first procedure has errors in comparison to experimental measurements, and the second procedure requires estimation of multiple per-compound physical fitting parameters (i.e., the Arrhenius pre-exponential constant *A*, the lateral diffusivity of the lipids, *D*_*L*_*(*T*)*, and the apparent transmembrane free energy apparent barrier height, *G*_*0*_). There are currently no known rapid ways to obtain a relative ranking of compounds crossing the BBB that are not based on estimates from structure–activity relation approximations [1, 13, 32–34].

In this study, we propose a method for determining quantitative transmembrane transport based on TBC rates obtained from a single equilibrium MD simulation, with a temperature-based enhancement of sampling that can perform relative ranking of permeability. This method leverages the recent advances in atomic scale modeling and computing power to overcome the limitations of current methods. The proposed method utilizes a single MD simulation at high temperature for a central nervous system (CNS) compound, which can be converged using a single graphics processing unit (GPU) node within a time frame of 24 to 72 h. Our approach is based on a least-squares fitting procedure that utilizes a library of CNS compounds of clinical importance with permeabilities spanning a broad range of interest, from the slowest range (10^–7^ cm/s) to fast CNS penetration (10^–4^ cm/s). We use a sample size of *N* = 18 CNS compounds which are chosen based on their clinical relevance and that their permeabilities span a broad range of interest. This approach provides a rapid and efficient way to determine the transmembrane transport of CNS compounds, and it could be useful for identifying promising lead compounds early in the drug development process.

## Materials and methods

### Single MD simulations for a library of compounds

#### Key MD simulation parameters

Unbiased atomistic MD simulations were performed using GROMACS (www.gromacs.org) [35] with the CHARMM36 all-atom force field for lipids [36], the CHARMM general force field (CGenFF) for molecular solutes and the TIP3P water model as solvent [37]. Electrostatic interactions were computed using particle-mesh-Ewald (PME) [38], and a cut-off of 10 Å was used for non-bonded interactions. Bonds involving hydrogen atoms were restrained using LINCS [39] to permit a 2 fs time-step. Neighbor lists were updated every five steps.

All simulations were performed in the NPT (constant N, pressure and temperature) ensemble, with water, lipids, and drug molecules coupled separately to a heat bath with temperatures at, respectively, 127 °C (simulation set 1), and 167 °C (simulation set 2). A time constant τ_T_ = 0.1 ps was utilized in combination with the velocity rescaling algorithm [40]. In the equilibration stage, an atmospheric pressure of 1 bar was maintained in the simulation box using the Berendsen semi-isotropic pressure coupling [41] with compressibility κ_z_ = κ_xy_ = 4.6 × 10^–5^ bar^−1^. In the production runs we utilized the Parrinello–Rahman semi-isotropic pressure coupling [42] with compressibility κ_z_ = κ_xy_ = 4.6 × 10^–5^ bar^−1^ and time constant τ_P_ = 20 ps. In order to capture diffusion events at sufficiently high resolution, trajectories were recorded every 1 ps, such that each 1 ms of trajectory comprises a dataset of 10^7^ observations.

#### A lipid bilayer model of human brain microvascular endothelial cells (BMECs)

An atomic detail molecular model of the apical BMEC lipid bilayer was utilized as described in previous work [24], consisting of a patch of 96 lipids (48 per leaflet) in an area of about 25 nm^2^. Information about the spatial lipid distribution within the individual leaflets was not available and was not incorporated in the model.

#### A compound library of the CNS drug space

A library of molecular solutes (N = 18; Table S1 and molecular structures in Figure S1) was chosen to be sufficiently broad and representative of CNS drugs, spanning a range of permeabilities (from 10^–7^ to 10^–3^ cm/s), charge (neutral, cationic, anionic, and zwitterionic), and lipophilicity (Log*P*_w/oct_ values from − 1.8 (polar) to 5.10 (non-polar)). Solute transport is dependent, in part, on the choice of solute force field [22, 43, 44]. Force field parameters were obtained in a standardized fashion using the CGenFF program [45, 46] (version 2.0) to obtain bonded and nonbonded parameters via the automated parameter assignment tool (Paramchem) [47] of CGenFF. The quality of similarity assignment for bonded and non-bonded parameters was monitored by penalty scores (Table S.4). All molecules are simulated in their neutral form, for comparison. The list of compounds is given in Fig. 1.

To converge the permeability estimates, simulations of solute transbilayer BBB crossing are carried out at temperatures greater than 25 °C (167 °C). This high-T MD methodology dramatically increases the rate of solute transport across lipid bilayers, and is applicable to lipid membranes in conjunction with small-molecule solutes that do not denature at T > 25 °C.

#### Simulations of solute translocation

The standard simulation system is different from that reported in our previous work by having an increased amount of water content. The system contains 7031 water molecules, 96 lipids, and 20 solute molecules distributed randomly with PACKMOL [48]. Simulations at elevated temperatures were necessary to enable accurate determination of the translocation frequency for solutes with low permeability. This high-T MD methodology is applicable to lipid membranes in conjunction with small-molecule solutes that do not denature at 167 °C ≥ T > 36.8 °C[24, 26]. The use of non-polarizable water models, such as TIP3P, at high temperatures carries limitations. While high-temperature simulations are well established in the field of MD simulation, the water models do not accurately describe the phase transitions associated with heating (or cooling). However, since we are not aiming to describe transport properties in water, we hold that the methodology is applicable to solute translocation across low dielectric media, such as a lipid bilayer.

#### Quantifying solute translocation across the lipid bilayer

There are two general approaches for calculating permeability in simulations: Fick’s law counting-based methods (used in this work), and methods based on the inhomogeneous solubility-diffusion (ISD) equation [19]. The ISD model is more complex since it requires accurate determination of the diffusion coefficient and free energy surface at each location (i.e. position in the bilayer), both of which are challenging [49–52]. Here we use direct observation from the simulations to identify individual translocation events.

The solute translocation frequency (*k*) across the bilayer is the number of translocation events per unit time. A translocation event is defined when a solute molecule moves from bulk solution on one side of the lipid bilayer, across the bilayer, and crosses a plane 1.0 nm beyond the bilayer on the opposite side. In the simulations, we define the steady state translocation k frequency as the average value when the derivative is less than a threshold value: i.e., dk/dt = [(k(i + 1) − k(i))/∆t] < 0.004.

### Free-energy surfaces (FES) and grouping of solutes

The FES profiles were calculated by first binning the *z*-positions of a solute to generate a 1-dimensional probability distribution *P*(*z*). The free-energy, *F*(*z*), was calculated by Boltzmann reweighting of the probability distribution (see *Supplementary Information* for details).

### Experimental values of permeability

Experimental values of permeability derived from the 2D transwell assay (*P*_app_) are widely used to predict brain penetration of small molecules, and the transwell assay is often considered the gold standard for validating simulations and in assessing barrier function of other in vitro models [53]. The most common cell lines are Madin–Darby Canine Kidney (MDCK) cells [16, 17], as well as Caco-2 cells with the PAMPA assay [54, 55]. We have chosen solute values of the cell line available for the compound of interest in published literature, with compounds missing a reference value measured as *in-house* measurements (Department of Materials Science & Engineering, Johns Hopkins). We have opted to use only *P*_app_ values as a benchmark, and not rat brain perfusion values *P*_3D_, due to the scarcity of data and the closer mimetics of our simulation system with the transwell assay. The final reference values are produced in Table S.1.

## Results

The passive BBB transport of small molecules involves crossing the luminal and abluminal membranes of BMECs in the cerebrovasculature (Fig. 1A). Previous work demonstrated high similarity in transport across the apical and basolateral chambers, so we chose to simulate the apical chamber only. As long as transport within the cell (between the two membranes) is fast in comparison to transport across the membranes, then the experimentally determined unidirectional diffusional permeability from the transwell assay is equal to half of the simulated permeability from the bidirectional flux, *P*_app_ = *P*_sim_/2, where *P*_sim_ is the permeability across a single bilayer (Fig. 1A). Here we do not include efflux pumps or other membrane proteins in the bilayer in order to focus on the effects of passive BBB translocation.

### Solute transport

MD simulations were performed at 167 °C for a library of solutes (*N* = 18; Fig. 1D) in a box (~ 5 × 5 × 12 nm) containing a BBB lipid bilayer model (Fig. 1B, C). Simulations were performed at elevated temperatures to enable a sufficient number (i.e. at least 1 and on average 10 events in 100 ns; Figure S1) of translocation events for accurate assessment of the permeability. The number of translocation events per unit time, *k*, is related to the permeability by: *P*_sim_ = r/2AC where r is the rate (*k*/N_A_), *A* is the lateral cross-sectional area of the membrane, and *C* is the concentration of the solute. The temperature required for efficient unbiased sampling is constrained, in large part, by the solutes with the lowest permeability, which require relatively long times to obtain accurate values of the rate constant. For the library of solutes reported here, each rate constant converged to a steady state value within 0.2–2.0 μs (Figures S1, S2), scaling at 0.1–0.2 μs per 24 h on a computing node with 4–8 computing processing units (CPUs) and GeForce GT × 1080 Ti GPU card.

**Fig. 2:**
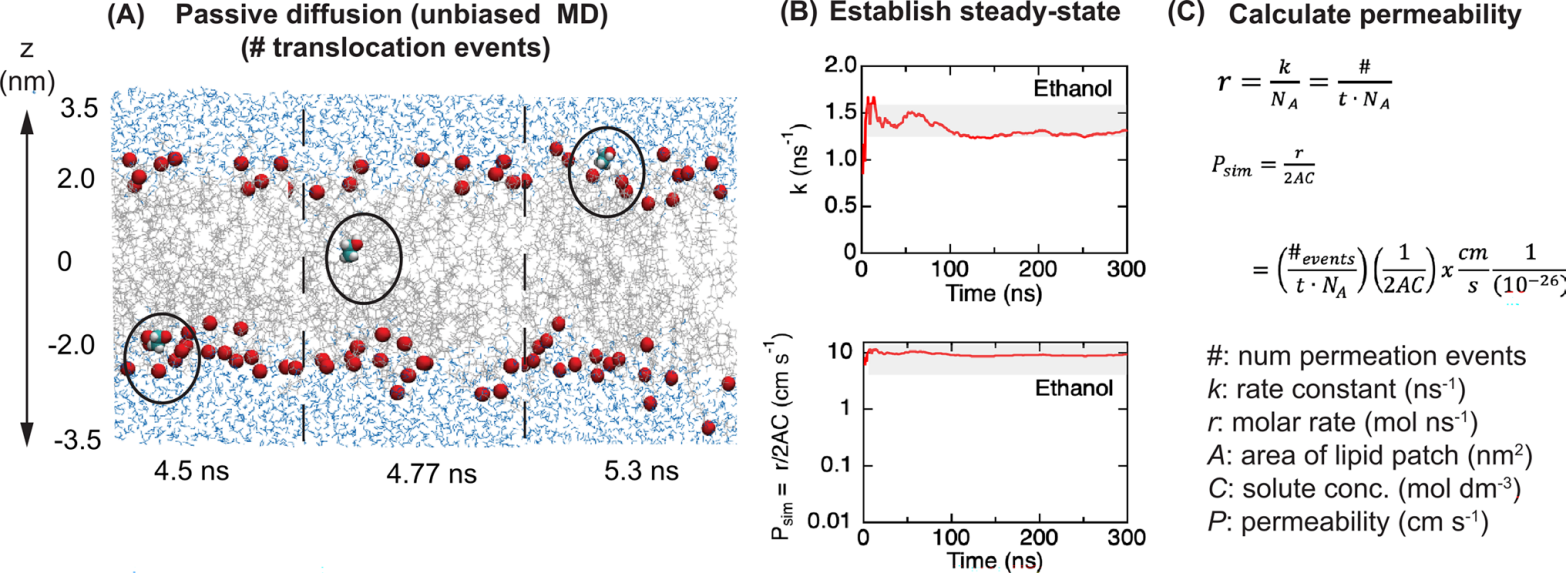
Obtaining membrane permeability (*P*_sim_) estimates from high-temperature MD simulations. For permeability estimates: **A** perform unbiased MD simulation of explicit atom transbilayer crossing at high temperature to obtain an estimate of the rate of crossing *k* based on the # of translocation events, **B** establish the steady state behaviour of the rate *k* (s), molar rate *r* (mol/s) to ensure the system has reached equilibrium, **C** calculate the permeability (cm/s)

As an example, we simulated the transport of ethanol (Fig. 2). Following simulation, we observe individual molecules crossing the bilayer over periods lasting ~ 1 ns (Fig. 2A). From the running time average of the number of crossings, a steady state in the rate constant, *k*, is reached after about 100 ns (using the plateau value from d*k*/d*t*), and from the plateau region, we determine a permeability of 8.9 × 10^0^ cm/s (Fig. 2B) using the procedure outlined in Methods (Fig. 2C).

The permeability values for all solutes at 167 °C are summarized in Table 1 (additional parameters for calculation are provided in Table S.5). The permeabilities *P*_sim_ span more than two orders of magnitude from 7 × 10^–2^ to 1.69 × 10^1^ cm/s. As expected, these values are much higher than the experimentally determined values at room temperature obtained from transwell measurements, which are typically in the range from 10^–7^ to 10^–3^ cm/s [56]. The temperature dependence on permeability can be inferred from the values of *P*_sim_ at 127 °C (Table 1), which are 5 to tenfold smaller than at 167 °C.

**Table 1:** Kinetic parameters extracted from simulations of the library of solutes (127, 167 °C) ordered alphabetically with a unique index (#)

Molecule index (#)	Molecule	*k* (ns^−1^) 127 °C	*k* (ns^−1^ ± σ) 167 °C	*P*_*sim*_ (× 10^–1^ cm/s ± σ) 127 °C	*P*_*sim*_ (× 10^−1^cm/s ± σ) 167 °C
1	Atenolol	0.010 ± 0.00	0.039 ± 0.00	0.77 ± 0.00	7.00 ± 0.36
2	Bupropion	0.010 ± 0.00	0.112 ± 0.00	0.74 ± 0.00	15.6 ± 0.56
3	Dilantin	0.025 ± 0.01	0.208 ± 0.01	1.84 ± 0.68	28.9 ± 1.39
4	Duloxetine	0.008 ± 0.00	0.032 ± 0.00	0.57 ± 0.00	4.50 ± 0.28
5	Effexor	0.016 ± 0.00	0.142 ± 0.00	1.21 ± 0.00	19.7 ± 0.28
6	Ethanol	0.410 ± 0.01	1.294 ± 0.03	30.8 ± 0.75	89.9 ± 1.81
7	Ibuprofen	0.012 ± 0.00	0.300 ± 0.01	0.90 ± 0.00	20.8 ± 0.69
8	Ketoprofen	0.028 ± 0.00	1.035 ± 0.02	2.12 ± 0.00	71.9 ± 1.39
9	Nadolol	0.016 ± 0.00	0.071 ± 0.01	1.20 ± 0.00	6.50 ± 0.91
10	Naproxen	0.022 ± 0.00	0.860 ± 0.03	1.65 ± 0.00	59.7 ± 2.08
11	Nicotine	0.335± 0.01	1.419 ± 0.02	25.1 ± 2.18	98.5 ± 1.11
12	Propanol	0.498 ± 0.01	2.425 ± 0.04	37.4 ± 0.75	168.4 ± 2.99
13	Ritalin	0.087 ± 0.00	0.654 ± 0.02	6.51 ± 0.00	45.4 ± 1.18
14	Caffeine	*–*	0.006 ± 0.00	*–*	0.90 ± 0.28
15	Doxorubicin	*–*	0.003 ± 0.00	*–*	0.70 ± 0.11
16	Ethosuximide	*–*	0.087 ± 0.01	*–*	7.10 ± 0.41
17	Glycerol	*–*	0.017 ± 0.01	*–*	1.20 ± 0.36
18	Temozolomide	*–*	0.018 ± 0.00	*–*	1.30 ± 0.15

Non-available entries due to lack of convergence are indicated as *NA (–)*. Measurement errors are provided as ± 1 standard deviation (σ)

**Fig. 3:**
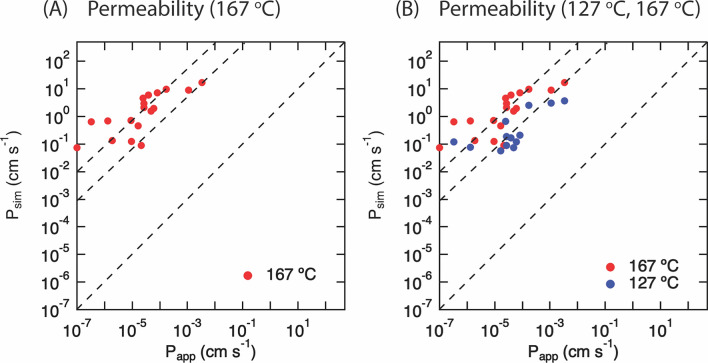
Permeabilities correlation (*P*_app_ vs *P*_sim_) for *N* = 18 compounds. **A** At 167 °C, depicting the roughly linear distribution of *P*_sim_/*P*_app_ along a line that is parallel to the equivalency line by a constant. The value and origin of this shift are confirmed in (**B**) for simulations at 127 °C and 167 °C

On comparing the values of permeability from the simulations (*P*_sim_) at 167 °C with experimental values (*P*_app_) recorded at ranges of at 25–37 °C (Fig. 3), with the predominant temperature being 25 °C and the following assay sources including Caco-2 cell lines as well as MDCK cell line measurements at 37 °C and red blood cell estimates using the PAMPA assay [57]. There is a reasonable correlation (*R*^2^ = 0.59) with an offset of about 5 orders of magnitude.

To verify the statistical significance of the sample size (*N* = 18), we perform independent regressions in sample sizes of incremental *N* (steps of *N* = 3 compounds; *N* = 3, 6, 9, 12, 15, 18 compounds). To remove bias, we perform combinatorial sampling to all possible combinations of points (816, 18,564, 48,620, 18,564, 816) in the dataset. The mean and standard deviation of the slopes of these regression lines were recorded and were used to assess the variability of the slopes. In Figure S3, we report the results of the search, which demonstrate that the average slope reaches a plateau beyond *N* = 12 compounds, suggesting that this method can be utilized for datasets above 12 compounds.

**Fig. 4:**
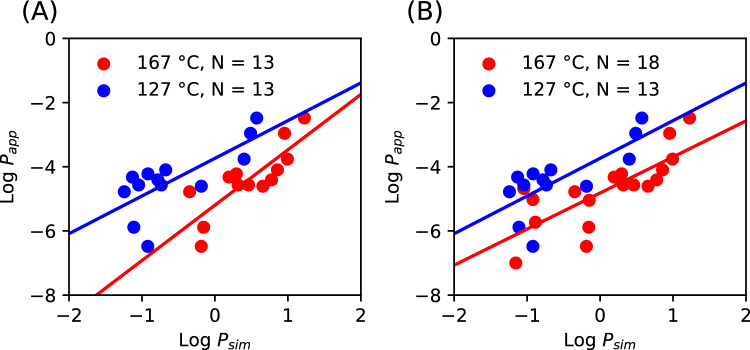
Least-squares fit of simulated permeability data and experimental permeability. **A** Least-squares fit regression of Log*P*_sim_ vs Log*P*_app_ with *N* = 13 compounds (167 °C, 127 °C) indicating the resulting regression y = mx + c. For 167 °C, m = 1.726, c = − 5.20, *R*^2^ = 0.68, *p*-value = 0.00054. For 127 °C, m = 1.17, c = − 3.73, *R*^2^ = 0.54, *p*-value = 0.0042. **B** Least-squares fit regression of Log*P*_sim_ vs Log*P*_app_ with *N* = 18 compounds (167 °C) and *N* = 13 (127 °C), indicating the resulting regression y = mx + c. For 167 °C, m = 1.124, c =  − 4.819, *R*^2^ = 0.59, *p*-value = 0.00021. For 127 °C, m = 1.17, c =  − 3.73, *R*^2^ = 0.54, *p*-value = 0.0042. Log denotes Log in base 10

In previous work [26], we outline computationally intensive ways to map the relationship between experimental and simulated permeabilities. We used the same library of compounds, with *N* = 18 converged at 167 °C and *N* = 13 converged at lower temperature. Here, we describe a relationship between the high-temperature simulated permeability and measured experimental permeability (*N* = 18 compounds) using a least-squares fit from the stats.linregress function of Scipy (Fig. 4) [58].

This allows for a relative ranking of novel compounds of interest to one order of magnitude of precision (Table 2; 167 °C; *R*^2^ of 0.59, *p* = 0.00021; Eq. 1; 127 °C; *R*^2^ of 0.54, p = 0.0042; Eq. 2). Specifically, using the fit we propose (Eq. 1; fit using 167 °C), one can rank a novel compound, ‘*X’*, using a single simulation at 167 °C (*P*_sim,x,167°C_) to obtain a relative apparent permeability ranking, *P*_app,X,37°C_ at 37 °C.

1 $${\text{Log}}P_{{{\text{app}},{\text{X}},{37}^\circ {\text{C}}}} = {1}.{12}\left[ {{\text{Log}}P_{{{\text{sim}},{\text{X}},{167}^\circ {\text{C}}}} } \right]{-}{4}.{82}$$

2 $${\text{Log}}P_{{{\text{app}},{\text{X}},{37}^\circ {\text{C}}}} = {1}.{17}\left[ {{\text{Log}}P_{{{\text{sim}},{\text{X}},{127}^\circ {\text{C}}}} } \right]{-}{3}.{73}$$

**Table 2:** Regression prediction

Validation dataset: Utilizing 167 °C regression to predict Papp,X,37°C (m = 1.124; c = − 4.891)			
P_app,Exp_ Experimental (cm/s)		Equation (1) Prediction (*P*_app,X,37°C_) cm/s	Order of magnitude error: |Log*P*_*app,*Exp_ − Log*P*_app,X,37°C_|
Sertraline	2.10 × 10^–6^	1.67 × 10^–6^	1.25000
Risperdal	3.00 × 10^–5^	5.24 × 10^–5^	0.24000
Diazepam	4.60 × 10^–5^	5.27 × 10^–5^	0.94000
Lacosamide	1.60 × 10^–5^	1.02 × 10^–4^	0.81000

Training dataset: Utilizing 167 °C regression to predict *P*_app,X,37°C_ (m = 1.124; c = − 4.891)				
Input for regression P_sim_(167 °C) (cm/s)	P_app,Exp_ Experimental (cm/s)		Equation (1) Prediction (*P*_app,X,37°C_) cm/s	Order of magnitude error: |Log*P*_*app,*Exp_ − Log*P*_app,X,37°C_|
8.99 × 10^0^	Ethanol	1.10 × 10^–3^	1.79 × 10^–4^	0.78837
9.85 × 10^0^	Nicotine	1.73 × 10^–4^	1.98 × 10^–4^	0.05958
1.56 × 10^0^	Bupropion	4.75 × 10^–5^	2.50 × 10^–5^	0.27862
2.89 × 10^0^	Dilantin	2.70 × 10^–5^	5.00 × 10^–5^	0.26769
9.00 × 10^–2^	Caffeine	2.10 × 10^–5^	1.01 × 10^–6^	1.31665
1.20 × 10^–1^	Glycerol	9.50 × 10^–6^	1.40 × 10^–6^	0.83172
1.30 × 10^–1^	Temozolomide	1.86 × 10^–6^	1.53 × 10^–6^	0.08444
7.00 × 10^–1^	Atenolol	1.30 × 10^–6^	1.02 × 10^–5^	0.89295
7.00 × 10^–2^	Doxorubicin	1.00 × 10^–7^	7.64 × 10^–7^	0.88289
Avg. Error: 0.55040				

Training dataset: Utilizing 127 °C regression to predict P_app_,_X_,_37°C_ (m = 1.277; c = − 3.65)				
Input for regression P_sim_(127 °C) (cm/s)	P_app,Exp_ Experimental (cm/s)		Equation (2) Prediction (*P*_app,X,37°C_) cm/s	Order of magnitude error: |Log*P*_*app,*Exp_ − Log*P*_app,X,37°C_|
3.73 × 10^0^	Propanol	3.30 × 10^–3^	8.69 × 10^–4^	0.57961
3.08 × 10^0^	Ethanol	1.10 × 10^–3^	6.94 × 10^–4^	0.20028
2.51 × 10^0^	Nicotine	1.73 × 10^–4^	5.47 × 10^–4^	0.49977
2.11 × 10^–1^	Ketoprofen	8.00 × 10^–5^	3.02 × 10^–5^	0.42368
7.40 × 10^–2^	Bupropion	4.75 × 10^–5^	8.85 × 10^–6^	0.72969
1.83 × 10^–1^	Dilantin	2.70 × 10^–5^	2.55 × 10^–5^	0.02430
5.72 × 10^–2^	Duloxetine	1.66 × 10^–5^	6.55 × 10^–6^	0.40395
7.73 × 10^–2^	Atenolol	1.30 × 10^–6^	9.31 × 10^–6^	0.85523
1.20 × 10^–1^	Nadolol	1.00 × 10^–7^	1.56 × 10^–5^	2.19264
Avg. Error: 0.46457				

For a range of simulated permeability (*P*_sim,x,167°C_) ordered by magnitude and output 37 °C relative permeability (*P*_app,X,37°C_) based on Eqs. 1 and 2 experimental permeability in Table S.1. Validation dataset denotes simulations not included in the regression model or anywhere else, while training set denotes values taken from within regression dataset.

The boundaries of the regression have been tested for a range of experimental permeabilities from 10^–7^ cm/s (doxorubicin) to 10^–3^ cm/s (ethanol) at two temperatures.

**Fig. 5:**
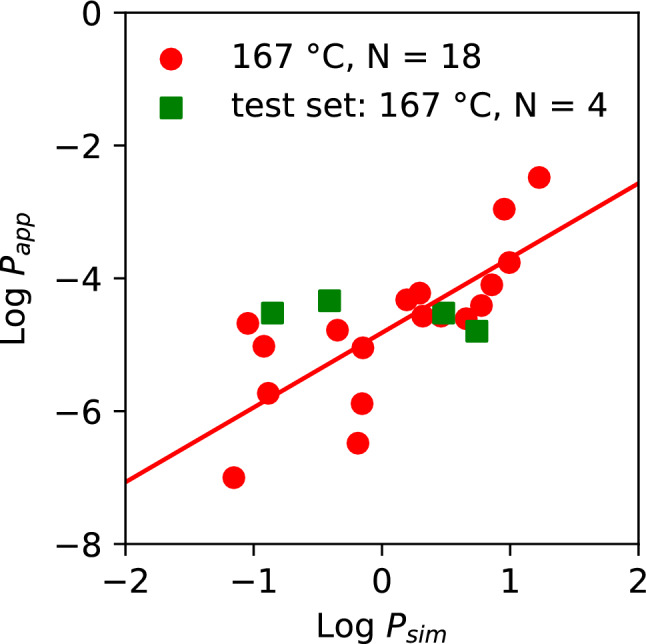
Least-squares regression using external dataset (*N* = 4). Red: Least-squares fit regression of Log*P*_sim_ vs Log*P*_app_ with *N* = 18 compounds (167 °C) and *N* = 13 (127 °C), indicating the resulting regression y = mx + c. For 167 °C, m = 1.124, c =  − 4.819, *R*^2^ = 0.59, *p*-value = 0.00021. For 127 °C, m = 1.17, c =  − 3.73, *R*^2^ = 0.54, *p*-value = 0.0042. Green: External verification set (*N* = *4)* not included in the regression set

In Table 2, we demonstrate the application of the regressions to perform a relative ranking of a novel compound X. For a compound with a 167 °C simulated permeability *P*_sim,X,167°C_ of 7 × 10^–2^ cm/s, this yields a predicted value of experimental permeability, *P*_app,X,37°C_, of 7.64 × 10^–7^ cm/s, while for a 167 °C permeability *P*_sim,X,167°C_ of 1.2 × 10^–1^ cm/s, this yields a value of *P*_app,X,37°C_ of 1.4 × 10^–6^ cm/s. Finally, for *P*_sim,X,167°C_ of 9.85 × 10^0^ cm/s, this yields a prediction for the value of *P*_app,X,37°C_ of 1.98 × 10^–4^ cm/s. The regression therefore spans the 4 orders of magnitude of *P*_app_ with significant predictive power. The error between the predicted experimental permeability (*P*_app,X,37°C_) and the known *in-vitro* permeability *P*_app_ is less than one order of magnitude (0.44–0.55; Table 2). To verify the model with an external dataset, we generated a test set outside the range of regression compounds (*N* = 4) using the same setup as depicted in the Methods section. In Fig. 5 we depict the results of applying regression (Eq. 1) to an external dataset (details in Table S7) of 167°C permeability. We find that the external test set lies within the range described by the regression, and thus the regression can be used to predict the experimental permeability for an unknown compound X from a single high-temperature simulation.

## Discussion

Understanding the mechanisms of drug transport across the BBB is critical for the development of therapeutics for CNS disorders. The BBB effectively restricts the entry of most molecules from the bloodstream into the CNS, making it difficult to deliver drugs to the brain [68]. A better understanding of the mechanisms of drug transport across the BBB will allow for the development of more effective therapeutics for the treatment of CNS disorders. As an example, several anti-amyloid antibodies (AAA) therapeutics for Alzheimer’s have failed to cross the BBB, thereby halting advanced stage clinical trials [69].

In this work, we have introduced a computational approach for ranking the permeability of a library of CNS compounds across the BBB. By simulating a library of interest at high temperature (T = 167 °C) and constructing a regression (Eq. 1) from the dataset of *P*_sim,167°C_ versus *P*_app_ it is possible to achieve relative prediction of an arbitrary compound’s permeability *P*_app,X,37°C_ from a single simulation (*P*_sim,x,167°C_). The method relies on efficient simulations at 167 °C that converge on a standard GPU typically in *t* ~ 24–72 h.

In the Results, we showed our simulated permeabilities for the compound library of solutes distribute with moderate linearity (Fig. 4), with a constant offset shift with respect to the experimental permeability (*P*_app_; 37 °C). When the temperature is lowered, the distribution shifts closer to the experimental permeability. Our methodology allows for the qualitative ranking of a compound permeability (*P*_app,X,37°C_) at physiological temperature. The error between the predicted permeability (*P*_app,X,37°C_) and the known *in-vitro* permeability *P*_app_ is less than one order of magnitude (0.44–0.55; Table 2), demonstrating an acceptable accuracy of our ranking approach.

In Table 3 we present the final comparison between experimental permeabilities reference values, to those predicted by the regression (Eq. 1) from a single high-T simulation (in [26]), to those from Arrhenius extrapolation from multiple high-T simulations. As can be seen, the regression prediction shows significantly closer agreement with experimental reference value compared to Arrhenius prediction. We had previously described how Arrhenius predictions suffered from non-linear effects, which can be corrected for (as in the work of Wang et al. [24]). In proposing this regression approach, we are arguing that scarce access to computing time should not preclude users from performing a qualitative ranking that is often less than one order of magnitude from the experimental reference. In our previous work, we noted how there can be marked disagreements between experimental sources (*P*_app_ from cell lines or *P*_3D_ from rat brain perfusion measurements), which are notably concentrated for certain groups of molecules (one group displaying statistically significant deviations of over 1.5 orders of magnitude. This should serve as a caution to examine the source of experimental data carefully before using as benchmark.

**Table 3:** Comparison of room-temperature permeabilities from (i) experiment, (ii) regression prediction and (iii) Arrhenius extrapolation from multiple high-T simulations

Molecule	*P*_app_ experimental (cm/s)	*P*_app_ References	Equation (1) Prediction (*P*_app,X,37°C_) cm/s	*P*_sim, 37°C_ (cm/s) Arrhenius extrapolation from [26]	Order of magnitude error: |Log*P*_*app,*Exp_ − Log*P*_app,X,37°C_|
Atenolol	1.30 × 10^–6^	[59] Adson (1995)	1.01 × 10^–5^	9.38 × 10^–6^	0.89
Bupropion	4.75 × 10^–5^	[56] Summerfield (2007)	2.48 × 10^–5^	1.18 × 10^–6^	0.28
Dilantin	2.70 × 10^–5^	[56] Summerfield (2007)	4.97 × 10^–5^	2.31 × 10^–5^	0.26
Duloxetine	1.66 × 10^–5^	[60] Hellinger (2012)	6.17 × 10^–6^	4.86 × 10^–7^	0.43
Effexor	6.00 × 10^–5^	[60] Hellinger (2012)	3.23 × 10^–5^	9.70 × 10^–6^	0.27
Ethanol	1.10 × 10^–3^	[57] Brahm (1983)	1.77 × 10^–4^	1.58 × 10^–1^	0.79
Ibuprofen	2.70 × 10^–5^	*IH*	3.44 × 10^–5^	1.45 × 10^–6^	0.10
Ketoprofen	8.00 × 10^–5^	[61] Sun (2002)	1.38 × 10^–4^	8.68 × 10^–5^	0.24
Nadolol	3.30 × 10^–7^	[62] Yamashita (2000)	9.31 × 10^–6^	1.25 × 10^–5^	1.45
Naproxen	3.90 × 10^–5^	[63] Pade (1998)	1.12 × 10^–4^	2.30 × 10^–5^	0.46
Nicotine	1.78 × 10^–4^	[64] Garberg (2005)	1.96 × 10^–4^	2.16 × 10^–1^	0.05
Propanol	3.30 × 10^–3^	[57] Brahm (1983)	3.58 × 10^–4^	3.06 × 10^–1^	0.97
Ritalin	2.47 × 10^–5^	[65] Yang (2016)	8.24 × 10^–5^	2.69 × 10^–3^	0.52
Caffeine	2.10 × 10^–5^	*IH*	1.01 × 10^–6^	3.27 × 10^–2^	1.32
Doxorubicin	1.00 × 10^–7^	[60] Hellinger (2012)	8.01 × 10^–7^	4.68 × 10^–2^	0.90
Ethosuximide	9.00 × 10^–6^	[56] Summerfield (2007)	1.03 × 10^–5^	2.40 × 10^–1^	0.06
Glycerol	9.50 × 10^–6^	[66] Shah (1989)	1.44 × 10^–6^	2.00 × 10^–2^	0.82
Temozolomide	1.86 × 10^–6^	[67] Avdeef (2012)	1.56 × 10^–6^	3.12 × 10^–2^	0.08

*IH* denotes in-house measurement. The order of magnitude absolute error is between (i) and (ii)

Although the *R*^2^ values obtained from regression are statistically significant (*p* = 0.0002 for T = 167 °C; *p* = 0.0042 for T = 127 °C), sources of error can be ascribed to, in particular, the use of CGenFF force field parameters in a consistent way without reoptimization. Previous attempts at ranking permeability with a fast methodology, such as implicit solvent assumption [43], have found smaller permeability *R*^2^ value in agreement when ranked (below 0.2), and described errors of up to 10 orders of magnitude between in silico prediction and experimental permeabilities. Our methodology has a statistically significant (*p* value = 0.0002) *R*^2^ value and lower error and is fast to calculate on consumer-grade GPU cards. This work opens up the possibility for routine ranking of CNS candidates on a large scale.

We now proceed to discuss the inherent methodological limitations from the simulations and experiments. Experimentally, the transwell assay is currently a very popular in vitro experimental method to source for permeability [53, 70]. The main limitation in its ability to accurately determine BBB permeability is to reproducibly obtain sources of hBMEC and other supporting cell types [71], and one solution has been to source the cells from human induced pluripotent stem cells (iPSCs). Our methodology has used the to-date best available experimental data [56, 67], but there are still notable differences in the epithelial cell lines utilized in the transwell assay (MDCK, Caco-2), which are different from brain endothelial cells (hBMECs). The passive permeability across the cell membrane is affected by the native size of cells. In general, epithelial cells are much thicker that brain endothelial cells. Thus, the permeability coefficients measured in epithelial cell-derived cell lines and hBMECs may be inherently different.

Similarly for computational methods such as MD simulations with high performance computing, there are limitations [19]. These reside primarily with their ability to obtain sufficient sampling statistics in order to converge metrics such as permeability estimates. Using either the ISD framework or our flux-based method, convergence at 310 K remains highly challenging, and for flux-based methods, computationally intractable for permeabilities < 10^–6^ cm/s [26]. Our MD model does not model the paracellular pathway, only the transcellular pathway. Among the other challenges of using MD simulations of CNS penetration is reconciling the choice of model with the experimental setup. To date, no simulation attempt is known to model the paracellular pathway, which is the transport of drugs across the tight junctions of the BBB. This is partly due to the complexities in building a cell–cell system to represent the paracellular pathway. The paracellular pathway is crucial for the overall transport of drugs across the BBB, and further research is needed to accurately model this pathway.

A deep understanding of the molecular mechanisms underlying BBB transport is crucial for the development of effective therapeutics for CNS disorders. Further research is needed to understand how these mechanisms are affected by disease states, such as BBB dysfunction in neurodegenerative and psychiatric disorders [4]. This will provide insights into how the BBB can be targeted to enhance drug delivery to the brain in these disorders, and how future simulation topologies can be developed to better capture these affects.

In addition to the scientific value of the proposed method, it is also important to consider the practicality, scalability and cost-effectiveness for routine use in drug development and discovery [72]. This method is easy to implement and is fast and cost-effective and it should be able to handle large sets of compounds, as is typically required in a drug discovery pipeline. The use of high-T methodology has a cost-saving component that is well documented [73], and the use of GPU technology is a further cost-saving measure that scales well. As a next step, the approach outlined here could be combined with a combinatorial chemistry array of compounds as input for unbiased presynthetic screening for a library of drugs for optimizing BBB penetration via chemical modification.

## Conclusion

We describe an advance in the development of the in silico design and optimization of CNS drugs. By using a single high-temperature simulation that requires between 24 and 72 h of user time on a conventional GPU computing node, our method predicts a relative ranking in CNS penetration for novel compounds using a dataset of simulated permeabilities. In the future, this technology can be applied to lower temperature simulations, pending suitable hardware advances, and can easily be integrated into a larger and more efficient platform accelerating drug discovery (e.g., to screen a library of compounds and find candidates that enhance brain exposure). In particular, the current framework is applicable to any atomically parameterizable chemistry, including peptides and other biologicals, which are not currently covered by many in silico techniques.

By simulating a library of interest at high temperature and constructing a regression from the dataset of *P*_sim,167C_ versus *P*_app_ it is possible to do relative permeability prediction rankings for arbitrary compounds compared to a dataset of simulated permeabilities. The user needs to input a single simulation at high-T to perform a ranking of the compound at 37 °C to get a fit with a mean error to experiment below one (0.44–0.55) order of magnitude.

The wealth of detailed information provided by atomic-resolution simulation trajectories can be used to identify lead compounds through high-throughput screening and pre-synthetically guide lead compound design for enhancing CNS exposure. The presented approach provides a rapid and efficient way to determine the transmembrane transport of CNS compounds and could be useful for identifying promising lead compounds early in the drug development process.

## Supplementary Information

Below is the link to the electronic supplementary material.

Supplementary file1 (PDF 2187 kb)

## Data Availability

The datasets generated during and/or analysed during the current study are available from the corresponding author on reasonable request.
