# Supplementary material for: A least-squares-fitting procedure for an efficient preclinical ranking of passive transport across the blood–brain barrier endothelium
Source: J Comput Aided Mol Des. 2023 Aug 12;37(11):537–49. doi: 10.1007/s10822-023-00525-1 (PMC10505096; doi:10.1007/s10822-023-00525-1)
Supplement: Supplementary file 1 — Supplementary file1 (PDF 2187 kb) [file 10822_2023_525_MOESM1_ESM.pdf]

Supplementary Material

A least-squares-fitting procedure for an efficient preclinical ranking of passive transport across the blood–brain barrier endothelium

Christian Jorgensen[1, 5*], Evan P. Troendle[2], Jakob P. Ulmschneider[4], Peter C. Searson[1,3], Martin B. Ulmschneider[2]

[1]Institute for NanoBioTechnology, Johns Hopkins University, Baltimore, Maryland, USA
[2]Department of Chemistry, King's College, London, UK
[3]Department of Materials Science and Engineering, Johns Hopkins University, Baltimore, Maryland, USA
[4] School of Physics and Astronomy, Shanghai Jiao Tong University, Shanghai, China.

Table of Contents

Materials and Methods

List of Tables

Table S1. Reference physicochemical parameters for a library of solutes. Two-dimensional (2D) cell culture terminology includes red blood cell (RBC), Madin-Darby Canine Kidney (MDCK) cells, human epithelial colorectal adenocarcinoma (Caco-2) cells and induced pluripotent stem cell (iPSC) line.

Table S2. Position of phosphorous (P) atom in the BBB bilayer. Error range is for average in lower and upper leaflets.

Table S3. Comparison of lipid lateral diffusivity and lipid area-per-lipid (APL) to reference values.

Table S4. Penalty scores from Paramchem.[1]

Table S5. Simulation parameters for calculating permeability. Rate constant k (ns^{-1}) where $r = k / N_A$. Total simulation box volume (V_{box}), bulk water volume (V_{water}), bilayer patch area (nm^2), number of solute molecules in the box, and solute concentration in bulk water (C = N / V_{water}). Finally, permeability P_{app} = r / (2 •A • C). Simulated permeabilities from Jorgensen et al. 2022 in [2].

[5] Current Address: Department of Chemistry, Aarhus University, Langelandsgade 140, 8000 Aarhus C, Denmark

Table S6. Least-squares fit regression of LogP_{sim} vs LogP_{app} with N = 18 compounds (167 °C) and N = 13 (127 °C), indicating the resulting regression y = mx + c. For 167 °C, m = 1.124, c = -4.819, R^2 = 0.59. For 127 °C, m = 1.17, c = -3.73, R^2 = 0.54.

List of Figures

Figure S1. Spontaneous trans-bilayer transition rate (*k*) convergence as a function of simulation time (ns) for a library of (N = 18) drugs. The $\#_{events}$ is defined as the number of times a molecule spontaneously crosses the BBB membrane from – 3 nm to + 3 nm or vice-versa ($\#_{Events} = \#_{Up} + \#_{Down}$) . The molar rate constant $r = k / N_A$ = (mol ns^{-1}). A plateau in *k*, indicating convergence of the estimate, is checked by the forward difference gradient reduced to a threshold of grad < 0.004.

Figure S2. Convergence threshold for the numerical evaluation of the rate constant *k* based on forward-difference gradient. The numerical gradient of *k* is defined as a forward differences estimate of grad = $(k(i + 1) - k(i)) / \Delta t$, where $\Delta t = t(i+1) - t(i)$. A hard convergence criterion is imposed, where *k* is deemed converged if and only if grad < 0.004.

Figure S3. Average slope of experimental permeability vs. simulated permeability (error bars ± σ) as a function of compound sample size (N compounds) demonstrating adequate sample size. (A) n = 1 for 1 random subsample iterated as a function of N = 3 compounds per point. (B) n = 10 random searches in size of 3 compounds. (C) All possible combinations (for 3, 6, 9, 12, 15 compounds), therefore a total search of 816, 18564, 48620, 18564, 816 (unbiased by random seed). We find that a plateau is established past N = 12 compounds, thus validating the sample size for our regression, and therefore, the methodology presented herein.

Materials and Methods (Extended)

Simulation details

Unbiased atomic detail molecular dynamics (MD) simulations were performed using GROMACS (www.gromacs.org)[13], in combination with the CHARMM general force field for molecular solutes and the TIP3P water model as solvent[14]. Lipids parameters were taken from the CHARMM36 all-atom force field[15]. Electrostatic interactions were computed using particle-mesh-Ewald (PME)[16], and a cut-off of 10 Å was used for the van der Waals interactions. Bonds involving hydrogen atoms were restrained using LINCS[17] to allow a 2 fs time-step. Neighbour lists were updated every 5 steps. All simulations were performed in the NPT ensemble, with water, lipids, and drug molecules coupled separately to a heat bath with temperatures at 167 °C using a time constant $\tau_T = 0.5$ ps in combination with the velocity rescaling algorithm[18]. Atmospheric pressure of 1 bar was maintained using Parrinello−Rahman semi-isotropic pressure coupling with compressibility $\kappa_z = \kappa_{xy} = 4.6 \cdot 10^{-5}$ bar^{-1} and time constant $\tau_P = 20$ ps[19]. In order to capture diffusion events at sufficiently high resolution, trajectories were printed every 1 ps, such that each 1 μs of trajectory comprises a dataset of 1 x 10^7 observations.

An atomic detail model of the BBB bilayer

An atomic detail molecular model of the apical hBMEC lipid bilayer was constructed by closely replicating physiological lipid compositions (**Figure** 1.A-B). The BBB bilayer model (96 lipids, area 25 nm^2) was set up using standard compositions for polarized endothelial cell membranes[3, 4], which contain a high content of sphingolipids (SM)[5] cholesterol (CH), and phosphatidylcholine (PC)[3, 4], as well as phosphatidylethanolamine (PE), phosphatidylinositol (PI) lipids (**Figure** 1.B)[6]. Each bilayer consists of 9 lipids: POPC (dark gray), OSM (red), SAPE (purple), SAPS (brown), SAPI (orange), cholesterol (green), SAPC (cyan), SOPE (black), SLPC (magenta). Atomic detail models were constructed using the CHARMM-GUI membrane builder. Both the average length of the hydrocarbon chain (18.4 carbon atoms) and number of double bonds (1.5) of the fatty acid tails were matched to equivalent experimental values (18.4 and 1.5, respectively)[3].

We validate the model by considering the following physical properties of the system as a function of temperature, firstly, the temperature-dependent bilayer thickness in the range of ≈ 40 Å between 25 °C and 200 °C (**Table S.2**), which is consistent with the thinning of bilayers at higher temperatures[34, 35]. Secondly, we show consistent observations of lipid lateral diffusivity for POPC and SOPE, as well as individual area-per-lipid (APL) measurements of POPC and SOPE to reference values (**Table S.3**). Finally, we monitor and quantify the temperature dependence of the diffusion coefficients and the area-per-lipid of individual lipids of the apical lipid bilayer, which is consistent with previous work on T-dependent MD simulations[35]. Physical bilayer parameters were monitored as an indication of bilayer stability with temperature, and were compared to experimental measurement, in particular, the average lipid diffusion coefficient in the plane of the membrane, D_L(T), and area-per-lipid (APL) are validated to reference parameters. The D_L(T) was calculated by averaging the diffusion of heavy atoms for all lipid species in the bilayer for lipids POPC, SAPE, SOPE and cholesterol.

A compound library of the BBB drug space

A library of molecular solutes ($N = 18$; **Table S.1** and structures in **Figure S.1**) was chosen to be sufficiently broad and representative of BBB crossing. The library spans a complete range

of permeabilities from 10^{-7} cm/s (slowest) to 10^{-3} cm/s (fastest), and comprises neutral, cationic, anionic as well as zwitterionic behaviour in solution, and with log P values from -1.8 (polar) to 5.10 (non-polar).

At pH = 7 the neutral molecules ($N = 8$) were glycerol, ethosuximide, ritalin, ethanol, propanol, effexor, bupropion, and temozolomide. The cationic ionized ($N = 4$) compounds were ibuprofen, ketoprofen, naproxen and dilantin. The anionic ionized compounds were caffeine, atenolol, nadolol, nicotine, duloxetine, and doxorubicin ($N = 6$). All molecules are simulated in their neutral form, for comparison.

To converge the permeability estimates, simulations of solute transbilayer BBB crossing are carried out at temperatures greater than 25 °C (167 °C). This high-T MD methodology dramatically increases the rate of solute transport across lipid bilayers, and is applicable to lipid membranes in conjunction with small-molecule solutes that do not denature at T > 25 °C.[7]

The value of simulated permeabilities depend, in part, on the choice of solute force field[8-10]. Force field parameters were obtained in a standardized fashion using the CGenFF program[11, 12] (version 2.0) to obtain bonded and nonbonded parameters via the automated parameter assignment tool (Paramchem)[13] of CGenFF.

1.1 Experimental *in vitro* permeabilities

Experimental values of permeability derived from the 2D transwell assay (P_{app}) are widely used to predict brain penetration of small molecules, and the transwell assay is often considered the gold standard for validating simulations and in assessing barrier function of other *in vitro* models.[14] The most common cell lines are Madin–Darby Canine Kidney (MDCK) cells,[15, 16] as well as Caco-2 cells with the PAMPA assay.[17, 18] We have chosen solute values of the cell line available for the compound of interest in published literature, with compounds missing a reference value measured as an *in-house* measurements (Department of Materials Science & Engineering, Johns Hopkins). Final reference values are produced in **Table S.1**.

Quantifying spontaneous transbilayer crossings

A translocation event is defined when a solute moves from the bulk aqueous phase on one side of the membrane to the bulk aqueous phase on the other side of the membrane. Translocations across the periodic boundaries are not included. Simulations were initiated with 40 randomly placed (*gmx insert-molecules*) molecules in the aqueous phase, equivalent to a system concentration of ~300 mM. Steady state values of the rate constant k were obtained by averaging values of k over the plateau region of the running average of k (**Figure S.1**) (i.e., once the change dk/dt was less than 0.004 (**Figure S.2**)).

The rate constant, k, across the bilayer is calculated from unbiased MD simulations of spontaneous trans-bilayer solute crossing as the ratio of the total number of transport events observed during a simulation by the simulation time:

$$k = \frac{\#}{t} \qquad \text{(Equation S1)}$$

Transport events are captured by tracking the progress of individual molecules through planes perpendicular to the bilayer normal located at either interface ($\# = \#_{up} + \#_{down}$). For practical purposes, the transport rate constant *k,* is redefined as the molar rate constant *r* :

$$r = \frac{k}{N_A} \qquad \text{(Equation S2)}$$

The accurate counting of transport event *i* ($\#_i$) is non-trivial. To that extent, we define planes for start and end positions for complete transitions. The planes (**Figure 2.A**) need to satisfy the following criteria: solutes are counted as crossing only when, (i) the transition originates in the bulk region ($z_i < -3.0$ nm or $z_i > 3.0$ nm), as well as, (ii) the solute trajectory concludes in the other bulk region of the box ($z_f > 3.0$ nm or $z_f < -3.0$ nm). This criterion ensures that counted events obey a strict distance cutoff criterion. Secondly, molecules that cross the box via the periodic boundary conditions are excluded from the count. Thirdly, incomplete crossings or non bulk-to-bulk transitions are excluded.

In order to ensure the concept of bulk-to-bulk transition is obeyed rigorously, we consider the physical properties of the system as a function of temperature. Bilayer thickness is temperature-dependent, with thickness range of ≈ 4.0 nm between 37 °C and 200°C. In addition, the solutes are not point particles, but have significant diameters along their principal axis (**Table S.1**; ranging between 0.5-2.0 nm). Thus, in order to be able to define events (#) rigorously, a cutoff criterion of -3.0 nm to 3.0 nm (range of 6.0 nm) is needed to accommodate these observations. Shorter, less rigid definitions of the cutoff (-2.0 nm to 2.0 nm) resulted in overcounting of transition events. Based on the position of the phosphorous groups of the lipid bilayer (**Table S.2**), solutes are therefore required to cross the bilayer from an origin ≥ 1.0 nm from the bilayer, enter and exit the bilayer, and transition ≥ 1.0 nm further ahead of the bilayer, in order to be counted.

The convergence criterion for the rate constant *k* is two-fold. Firstly, we check for plateau in the value of *k* for each solute (**Figure S.1**), and second, we calculate the numerical gradient from a forward difference expression (Equation S3). The value of *k* is converged when the gradient descends below 0.004 (grad(*k*) < 0.004) (**Figure S.2**).

$$Grad(k) = \frac{k(i+1)-k(i)}{\Delta t} \qquad \text{(Equation S3)}$$

The solute permeability *P* (cm s^{-1}) is related to the net solute flux *J* (# cm^{-2} s^{-1}) through a membrane patch by:

$$J = P \cdot \Delta C \qquad \text{(Equation S4)}$$

Where $\Delta C = C_o - C_i$, and C_i (# m^{-3}), C_o (# m^{-3}) are the concentrations on either side of the membrane. Due to pressure coupling, the box volume, V, and area of the bilayer patch, A, will vary during the simulation and need to be averaged.

The permeability is obtained from equation 4, by calculation the flux *J*. The flux is calculated as the ratio of molar rate *r* (mol s^{-1}) per unit area (*A*; nm^2):

$$J = \frac{r}{A} \qquad \text{(Equation S5)}$$

This leads to a permeability expression of $P = r / AC$. Two caveats are introduced to obtain a final expression for the permeability. However, to be able to compare to experimental measurements, the total flux is divided by 2 as the simulations capture bi-directional flux, such that flux equals the upward plus downward flux ($J = J_{up} + J_{down}$). This results in the final expression for permeability (cm s^{-1}) as:

$$P_{sim} = \frac{r}{2AC} \quad \text{(Equation S6)}$$

It should be noted that these assumptions are different from experimental calculations, firstly, because *in vitro* assays measure flux in one direction only, and secondly, for *in silico* calculations, we assume that the transport via the cytosol (**Figure 1.A**) is fast compared to transport across the cell membrane:

$$k_{BBB} \ll k_{cytosol} \quad \text{(Equation S7)}$$

Note that at 127 °C, simulations for four of the solutes did not converge, necessitating higher temperature to compare relative permeability over this timescale.

List of Tables

Table S1. Reference physicochemical parameters for a library of solutes. Two-dimensional (2D) cell culture terminology includes red blood cell (RBC), Madin-Darby Canine Kidney (MDCK) cells, human epithelial colorectal adenocarcinoma (Caco-2) cells and induced pluripotent stem cell (iPSC) line. IH denotes in-house measurement (Department of Materials Science & Engineering, Johns Hopkins).

Molecule	MW (g mol^{-1})	Diameter (Å)	H_{donor}	H_{accept}	Log *P*	DP (debye)	Polar surf. area	P_{app} (cm s^{-1})	P_{app} Reference	Cell line (2D) or method (3D)
Glycerol	92.09	5.3	3	3	-1.8	2.62	60.7	9.50 x 10^{-6}	[19] [Shah 1989]	BMEC [36.9 °C]
Temozolomide	194.1	8.7	1	5	0.4	6.10	106.0	1.86 x 10^{-6}	[20] [Avdeef 2012]	PAMPA [25 °C]
Caffeine	194.2	6.4	0	3	-0.07	3.64	58.4	2.10 x 10^{-5}	*IH*	MDCK [25 °C]
Ethanol	46.1	2.7	1	1	-0.31	1.69	20.2	1.10 x 10^{-3}	[21] [Brahm 1983]	RBC [25 °C]
Propanol	60.1	4.1	1	1	0.05	1.68	20.2	3.30 x 10^{-3}	[21] [Brahm 1983]	RBC [25 °C]
Doxorubicin	543.5	13.6	6	12	1.27	9.12	206	1.00 x 10^{-7}	[22] [Hellinger 2012]	Caco-2 / MDCK [36.9 °C]
Ethosuximide	141.2	6.6	1	2	0.38	1.72	46.2	9.00 x 10^{-6}	[23] [Summerfield 2007]	MDCK [36.9 °C]
Atenolol	266.3	16.3	3	4	0.16	5.00	84.6	1.30 x 10^{-6}	[24] [Adson 1995]	Caco-2 [36.9 °C]
Nadolol	309.4	15.2	4	5	0.81	5.10	82.0	3.30 x 10^{-7}	[25] [Yamashita 2000]	Caco-2 [25 °C]
Dilantin	252.3	5.8	2	2	2.47	2.73	58.2	2.70 x 10^{-5}	[23] [Summerfield 2007]	MDCK [36.9 °C]
Ketoprofen	254.3	12.2	1	3	3.12	4.44	54.4	8.00 x 10^{-5}	[26] [Sun 2002]	Caco-2 [25 °C]
Naproxen	230.3	11.6	1	3	3.18	2.25	46.5	3.90 x 10^{-5}	[27] [Pade 1998]	Caco-2 [25 °C]
Nicotine	162.2	6.9	0	2	1.17	1.64	16.1	1.78 x 10^{-4}	[28] [Garberg 2005]	Caco-2 / MDCK [25 °C]
Ibuprofen	206.3	8.1	1	2	3.97	1.64	37.3	2.70 x 10^{-5}	*IH*	MDCK [25 °C]
Effexor	277.0	9.6	1	3	3.2	3.33	32.7	6.00 x 10^{-5}	[22] [Hellinger 2012]	Caco-2 / MDCK [36.9 °C]
Ritalin	233.1	7.3	1	3	2.25	2.13	38.3	2.47 x 10^{-5}	[29] [Yang 2016]	MDCK [36.9 °C]
Duloxetine	297.4	11	1	3	4.00	2.18	49.5	1.66 x 10^{-5}	[22] [Hellinger 2012]	Caco-2 / MDCK [36.9 °C]
Bupropion	239.7	8.3	2	4	3.60	1.15	29.1	4.75 x 10^{-5}	[23] [Summerfield 2007]	MDCK [36.9 °C]

Table S2. Position of phosporous (P) atom in the BBB bilayer. Error range is for average in lower and upper leaflets. From [2].

	T (°C)	Avg P position (nm)	Error range (σ) (nm)
Standard box	167	± 1.92	0.05-0.2
	25	± 2.22	0.04-0.14
Double box	167	± 1.97	0.2-0.3
	25	± 2.24	0.08-0.12

Table S3. Comparison of lipid lateral diffusivity and lipid area-per-lipid (APL) to reference values. From [2]

Property	Simulation	Reference
POPC Diffusivity (10^{-8} cm^2/s)	3.15 ± 0.8	3.2 - 5.68‡
SOPE Diffusivity (10^{-8} cm^2/s)	11.5 ± 7.3	2.0 – 4.0†
POPC APL (nm^2)	0.77 ± 0.05	0.643*
SOPE APL (nm^2)	0.62 ± 0.02	0.590*
CHOL APL (nm^2)	0.39 ± 0.04	0.393*

Table S4. Penalty scores from Paramchem.[1]

Compound	Param penalty	Charge penalty
Atenolol	43	26.6
Bupropion	43.3	38
Dilantin	115.3	132
Duloxetine	65.6	166.6
Effexor	18.3	34
Ethanol	0	0
Ibuprofen	14.4	46
Ketoprofen	14.3	46
Nadolol	34.5	36.9
Naproxen	14.3	46
Nicotine	77.2	118.5
Propanol	0	0
Ritalin	19.9	43
Doxorubicin	29.7	57
Caffeine	103.4	39.4
Ethosuximide	0	0
Glycerol	0	0
Temozolomide	176.5	378.5

Table S5. Simulation parameters for calculating permeability. Rate constant k (ns^{-1}) where $r = k / N_A$. Total simulation box volume (V_{box}), bulk water volume (V_{water}), bilayer patch area (nm^2), number of solute molecules in the box, and solute concentration in bulk water ($C = N / V_{water}$). Finally, permeability $P_{app} = r / (2 \bullet A \bullet C)$. Simulated permeabilities from Jorgensen et al. 2022 in [2] also provided.

Molecule	k ($ns^{-1} \pm \sigma$) 167 °C	Volume$_{box}$ (nm^3)	Volume$_{water}$ (nm^3)	A (nm^2)	N molecules	C (mol dm^{-3})	P_{sim} ($\times 10^{-1}$cm $s^{-1} \pm \sigma$) 167 °C	P_{sim} ($\times 10^{-1}$cm $s^{-1} \pm \sigma$) 167 °C [2]
Atenolol	0.039 ± 0.00	2.87×10^2	1.23×10^2	3.39×10^1	10	1.35×10^{-1}	7.00 ± 0.36	6.99
Bupropion	0.112 ± 0.00	3.80×10^2	2.00×10^2	3.60×10^1	20	1.66×10^{-1}	15.6 ± 0.56	8.92
Dilantin	0.208 ± 0.01	3.80×10^2	2.00×10^2	3.60×10^1	20	1.66×10^{-1}	28.9 ± 1.39	12.3
Duloxetine	0.032 ± 0.00	3.80×10^2	2.00×10^2	3.60×10^1	20	1.66×10^{-1}	4.50 ± 0.28	14.1
Effexor	0.142 ± 0.00	3.80×10^2	2.00×10^2	3.60×10^1	20	1.66×10^{-1}	19.7 ± 0.28	6.49
Ethanol	1.294 ± 0.03	3.80×10^2	2.00×10^2	3.60×10^1	40	3.32×10^{-1}	89.9 ± 1.81	99.9
Ibuprofen	0.300 ± 0.01	3.80×10^2	2.00×10^2	3.60×10^1	40	3.32×10^{-1}	20.8 ± 0.69	16.0
Ketoprofen	1.035 ± 0.02	3.80×10^2	2.00×10^2	3.60×10^1	40	3.32×10^{-1}	71.9 ± 1.39	27.2
Nadolol	0.071 ± 0.01	2.87×10^2	1.23×10^2	3.39×10^1	20	2.70×10^{-1}	6.50 ± 0.91	6.48
Naproxen	0.860 ± 0.03	3.80×10^2	2.00×10^2	3.60×10^1	40	3.32×10^{-1}	59.7 ± 2.08	33.7
Nicotine	1.419 ± 0.02	3.80×10^2	2.00×10^2	3.60×10^1	40	3.32×10^{-1}	98.5 ± 1.11	121.0
Propanol	2.425 ± 0.04	3.80×10^2	2.00×10^2	3.60×10^1	40	3.32×10^{-1}	168.4 ± 2.99	112.0
Ritalin	0.654 ± 0.02	3.80×10^2	2.00×10^2	3.60×10^1	40	3.32×10^{-1}	45.4 ± 1.18	17.7
Caffeine	0.006 ± 0.00	3.80×10^2	2.00×10^2	3.60×10^1	20	1.66×10^{-1}	0.90 ± 0.28	0.66
Doxorubicin	0.003 ± 0.00	3.80×10^2	2.00×10^2	3.60×10^1	10	8.31×10^{-2}	0.70 ± 0.11	0.63
Ethosuximide	0.087 ± 0.01	2.43×10^2	1.04×10^2	3.19×10^1	20	3.19×10^{-1}	7.10 ± 0.41	7.09
Glycerol	0.017 ± 0.01	3.80×10^2	2.00×10^2	3.60×10^1	40	3.32×10^{-1}	1.20 ± 0.36	1.01
Temozolomide	0.018 ± 0.00	2.20×10^2	9.43×10^1	3.19×10^1	20	3.52×10^{-1}	1.30 ± 0.15	1.32

Table S6. Least-squares fit regression of LogP_{sim} vs LogP_{app} with N = 18 compounds (167 °C) and N = 13 (127 °C), indicating the resulting regression y = mx + c. For 167 °C, m = 1.124, c = -4.819, R^2 = 0.59. For 127 °C, m = 1.17, c = -3.73, R^2 = 0.54.

	Compound	P_sim_127	P_sim_167	LogPsim127	LogPsim167	P_app
0	Atenolol	0.077303	0.70	-1.111803	-5.886057	1.300000e-06
1	Bupropion	0.074076	1.56	-1.130323	0.193125	4.750000e-05
2	Dilantin	0.183876	2.89	-0.735474	0.460898	2.700000e-05
3	Duloxetine	0.057264	0.45	-1.242116	-0.346787	1.660000e-05
4	Effexor	0.120833	1.97	-0.917815	0.294466	6.000000e-05
5	Ethanol	3.077113	8.99	0.488143	0.953760	1.100000e-03
6	Ibuprofen	0.090062	2.08	-1.045459	0.318063	2.700000e-05
7	Ketoprofen	0.211645	7.19	-0.674391	0.856729	8.000000e-05
8	Nadolol	0.120082	0.65	-0.920520	-0.187087	3.300000e-07
9	Naproxen	0.165113	5.97	-0.782218	0.775974	3.900000e-05
10	Nicotine	2.511224	9.85	0.399885	0.993436	1.730000e-04
11	Propanol	3.737566	16.84	0.572589	1.226342	3.300000e-03
12	Ritalin	0.651447	4.54	-0.186121	0.657056	2.470000e-05
13	Glycerol	NaN	0.12	NaN	-0.920819	9.500000e-06
14	Temozolomide	NaN	0.13	NaN	-0.886057	1.860000e-06
15	Caffeine	NaN	0.09	NaN	-1.045757	2.100000e-05
16	Doxorubicin	NaN	0.07	NaN	-1.154902	1.000000e-07
17	Ethosuximide	NaN	0.71	NaN	-0.148742	9.000000e-06

167 °C: [m = 1.124465920916853, c = -4.818930501473413, R^2 = 0.59]

127 °C: [m = 1.174649568849751, c = -3.7374618317313706, R^2 = 0.54]

List of Figures

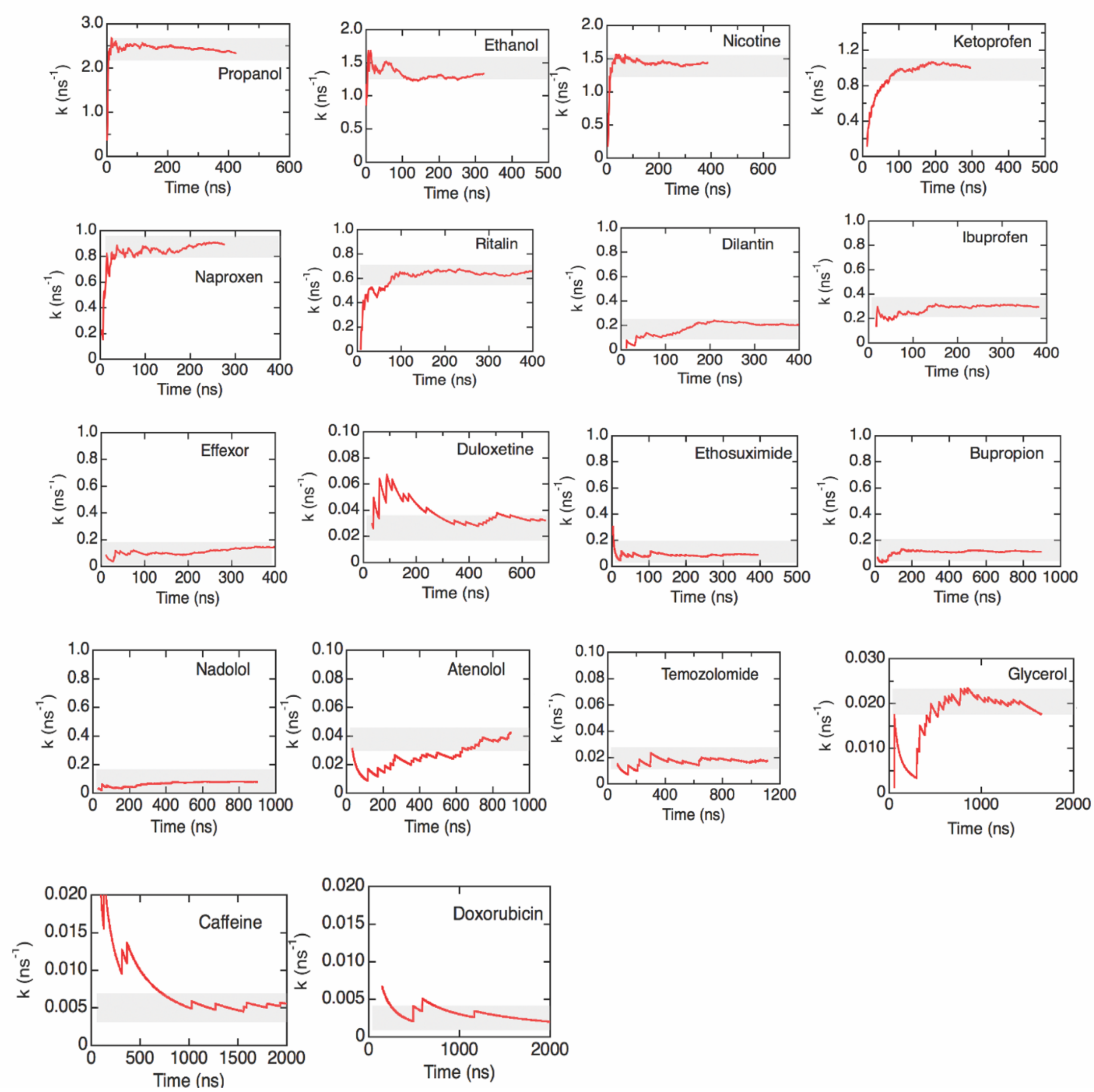

Figure S1. Spontaneous trans-bilayer transition rate (*k*) convergence as a function of simulation time (ns) for a library of (*N* = 18) drugs. The $\#_{events}$ is defined as the number of times a molecule spontaneously crosses the BBB membrane from – 3 nm to + 3 nm or vice-versa ($\#_{Events} = \#_{Up} + \#_{Down}$) . The molar rate constant $r = k / N_A$ = (mol ns^{-1}). A plateau in *k,* indicating convergence of the estimate, is checked by the forward difference gradient reduced to a threshold of grad < 0.004.

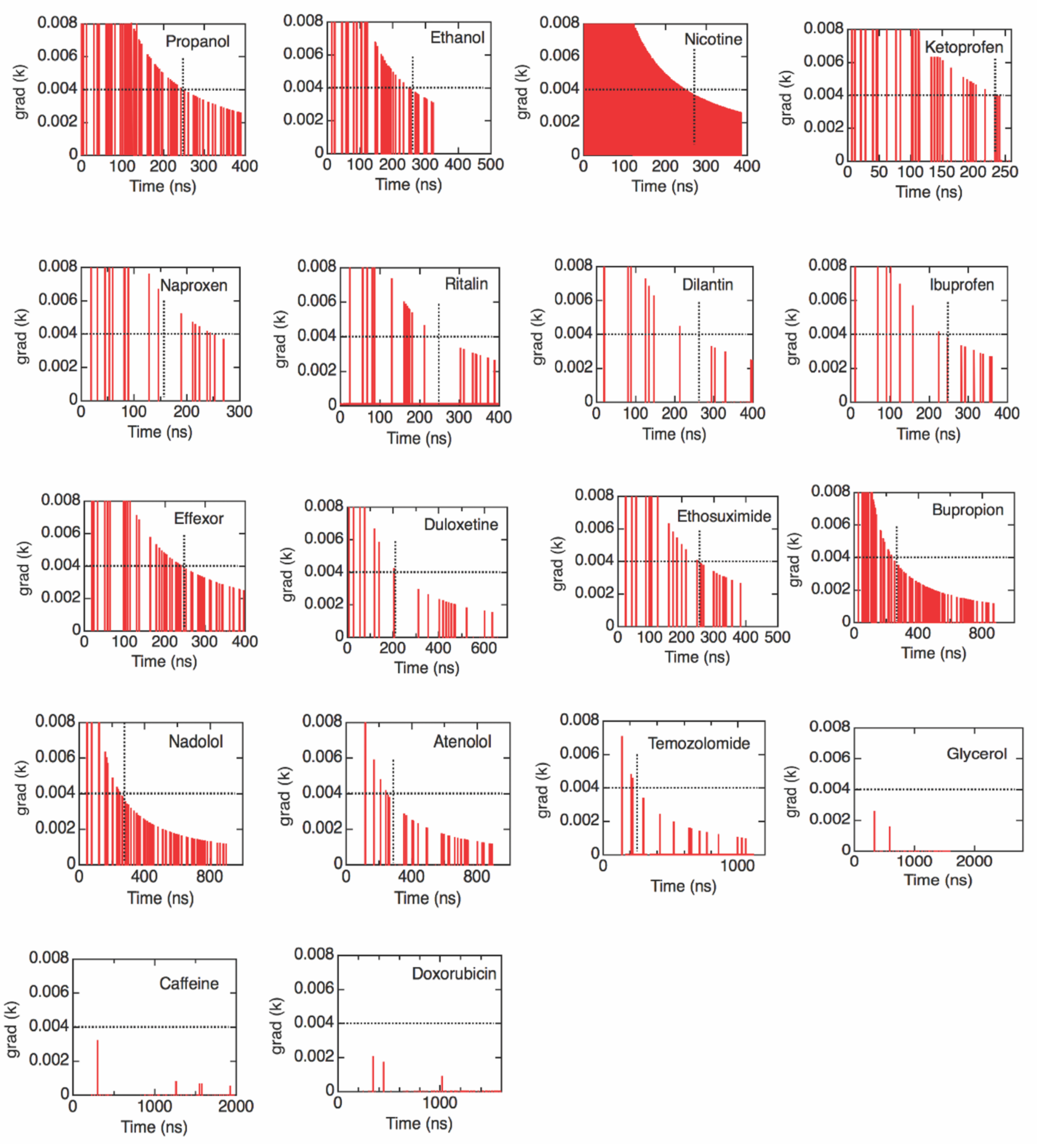

Figure S2. Convergence threshold for the numerical evaluation of the rate constant *k* based on forward-difference gradient. The numerical gradient of k is defined as a forward differences estimate of grad = $(k(i + 1) - k(i)) / \Delta t$, where $\Delta t = t(i+1) - t(i)$. A hard convergence criterion is imposed, where k is deemed converged if and only if grad < 0.004.

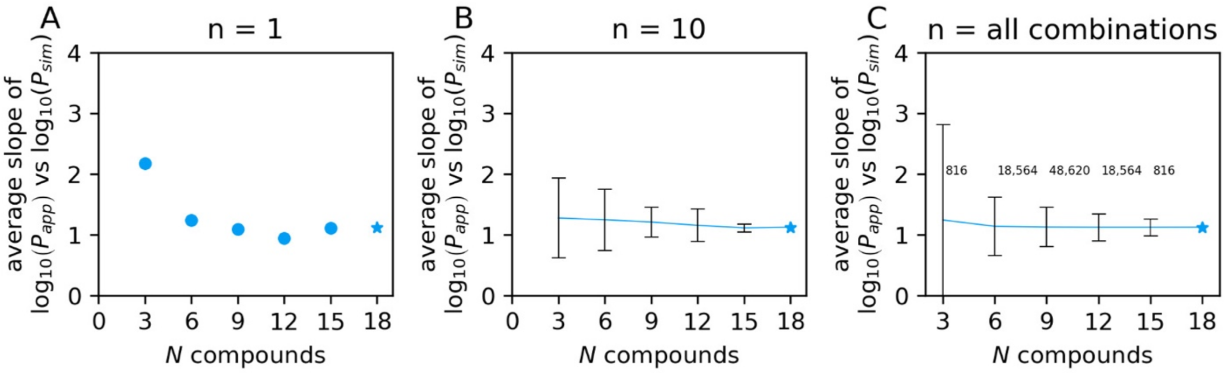

Figure S3. Average slope of experimental permeability vs. simulated permeability (error bars ± σ) as a function of compound sample size (*N* compounds) demonstrating adequate sample size. (A) n = 1 for 1 random subsample iterated as a function of $N = 3$ compounds per point. (B) n = 10 random searches in size of 3 compounds. (C) All possible combinations (for 3, 6, 9, 12, 15 compounds), therefore a total search of 816, 18564, 48620, 18564, 816 (unbiased by random seed). We find that a plateau is established past $N = 12$ compounds, thus validating the sample size for our regression, and therefore, the methodology presented herein.

References

1. Ghosh, J.; Marru, S.; Singh, N.; Vanomesslaeghe, K.; Fan, Y.; Pamidighantam, S., Molecular parameter optimization gateway (ParamChem): workflow management through TeraGrid ASTA. *Proceedings of the 2011 TeraGrid conference: extreme digital discovery* **2011**.
2. Jorgensen, C.; Ulmschneider, M. B.; Searson, P. C., Atomistic Model of Solute Transport across the Blood–Brain Barrier. *ACS omega* **2022,** *7*, 1100-1112.
3. Spector, A. A.; Hoak, J. C.; Fry, G. L.; Denning, G. M.; Stoll, L.; Smith, J., Effect of fatty acid modification on prostacyclin production by cultured human endothelial cells. *The Journal of clinical investigation* **1980,** *65* (5), 1003-1012.
4. Spector, A. A.; Yorek, M. A., Membrane lipid composition and cellular function. *Journal of lipid research* **1985,** *26* (9), 1015-1035.
5. Marsh, D., CRC handbook of lipid bilayers. **1990**.
6. Tewes, B.; Galla, H.-J., Lipid polarity in brain capillary endothelial cells. *Endothelium* **2001,** *8* (3), 207-220.
7. Wang, Y.; Gallagher, E.; Jorgensen, C.; Troendle, E. P.; Hu, D.; Searson, P. C.; Ulmschneider, M. B., An experimentally validated approach to calculate the blood-brain barrier permeability of small molecules. *Scientific reports* **2019,** *9* (1), 1-11.
8. Brocke, S. A.; Degen, A.; MacKerell Jr, A. D.; Dutagaci, B.; Feig, M., Prediction of Membrane Permeation of Drug Molecules by Combining an Implicit Membrane Model with Machine Learning. *Journal of chemical information and modeling* **2018**.
9. Menichetti, R.; Kanekal, K. H.; Bereau, T., Drug–Membrane Permeability across Chemical Space. *ACS Central Science* **2018**.
10. Dickson, C. J.; Hornak, V.; Bednarczyk, D.; Duca, J. S., Using membrane partitioning simulations to predict permeability of forty-nine drug-like molecules. *J. Chem. Inf. Mod.* **2018,** *59* (1), 236-244.
11. Vanommeslaeghe, K.; MacKerell, A. D., Jr., Automation of the CHARMM General Force Field (CGenFF) I: bond perception and atom typing. *J. Chem. Inf. Model.* **2012,** *52* (12), 3144-54.
12. Vanommeslaeghe, K.; Raman, E. P.; MacKerell, A. D., Jr., Automation of the CHARMM General Force Field (CGenFF) II: assignment of bonded parameters and partial atomic charges. *J. Chem. Inf. Model.* **2012,** *52* (12), 3155-68.
13. Ghosh, J.; Marru, S.; Singh, N.; Vanomesslaeghe, K.; Fan, Y.; Pamidighantam, S., Molecular parameter optimization gateway (ParamChem): workflow management through TeraGrid ASTA. **2011**.
14. Cecchelli, R.; Berezowski, V.; Lundquist, S.; Culot, M.; Renftel, M.; Dehouck, M.-P.; Fenart, L., Modelling of the blood–brain barrier in drug discovery and development. *Nature reviews Drug discovery* **2007,** *6* (8), 650.
15. Artursson, P.; Palm, K.; Luthman, K., Caco-2 monolayers in experimental and theoretical predictions of drug transport. *Adv. Drug Del. Rev.* **2012,** *64*, 280-289.
16. van Breemen, R. B.; Li, Y., Caco-2 cell permeability assays to measure drug absorption. *Expert opinion on drug metabolism & toxicology* **2005,** *1* (2), 175-185.
17. Avdeef, A., The rise of PAMPA. *Expert opinion on drug metabolism & toxicology* **2005,** *1* (2), 325-342.
18. Di, L.; Kerns, E. H.; Bezar, I. F.; Petusky, S. L.; Huang, Y., Comparison of blood–brain barrier permeability assays: in situ brain perfusion, MDR1-MDCKII and PAMPA-BBB. *Journal of pharmaceutical sciences* **2009,** *98* (6), 1980-1991.

19. Shah, M. V.; Audus, K. L.; Borchardt, R. T., The application of bovine brain microvessel endothelial-cell monolayers grown onto polycarbonate membranes in vitro to estimate the potential permeability of solutes through the blood–brain barrier. *Pharmaceutical research* **1989,** *6* (7), 624-627.
20. Avdeef, A., *Absorption and drug development: solubility, permeability, and charge state*. John Wiley & Sons: 2012.
21. Brahm, J., Permeability of human red cells to a homologous series of aliphatic alcohols. Limitations of the continuous flow-tube method. *J. Gen. Physiol.* **1983,** *81* (2), 283-304.
22. Hellinger, É.; Veszelka, S.; Tóth, A. E.; Walter, F.; Kittel, Á.; Bakk, M. L.; Tihanyi, K.; Háda, V.; Nakagawa, S.; Duy, T. D. H., Comparison of brain capillary endothelial cell-based and epithelial (MDCK-MDR1, Caco-2, and VB-Caco-2) cell-based surrogate blood–brain barrier penetration models. *Eur. J. Pharm. Biopharm* **2012,** *82* (2), 340-351.
23. Summerfield, S. G.; Read, K.; Begley, D. J.; Obradovic, T.; Hidalgo, I. J.; Coggon, S.; Lewis, A. V.; Porter, R. A.; Jeffrey, P., Central nervous system drug disposition: the relationship between in situ brain permeability and brain free fraction. *Journal of Pharmacology and Experimental Therapeutics* **2007,** *322* (1), 205-213.
24. Adson, A.; Burton, P. S.; Raub, T. J.; Barsuhn, C. L.; Audus, K. L.; Ho, N. F., Passive diffusion of weak organic electrolytes across Caco-2 cell monolayers: Uncoupling the contributions of hydrodynamic, transcellular, and paracellular barriers. *Journal of pharmaceutical sciences* **1995,** *84* (10), 1197-1204.
25. Yamashita, S.; Furubayashi, T.; Kataoka, M.; Sakane, T.; Sezaki, H.; Tokuda, H., Optimized conditions for prediction of intestinal drug permeability using Caco-2 cells. *Eur. J. Pharm. Sci.* **2000,** *10* (3), 195-204.
26. Sun, D.; Lennernas, H.; Welage, L. S.; Barnett, J. L.; Landowski, C. P.; Foster, D.; Fleisher, D.; Lee, K.-D.; Amidon, G. L., Comparison of human duodenum and Caco-2 gene expression profiles for 12,000 gene sequences tags and correlation with permeability of 26 drugs. *Pharmaceutical research* **2002,** *19* (10), 1400-1416.
27. Pade, V.; Stavchansky, S., Link between drug absorption solubility and permeability measurements in Caco-2 cells. *Journal of pharmaceutical sciences* **1998,** *87* (12), 1604-1607.
28. Garberg, P.; Ball, M.; Borg, N.; Cecchelli, R.; Fenart, L.; Hurst, R.; Lindmark, T.; Mabondzo, A.; Nilsson, J.; Raub, T., In vitro models for the blood–brain barrier. *Toxicol. In Vitro* **2005,** *19* (3), 299-334.
29. Yang, X.; Duan, J.; Fisher, J., Application of physiologically based absorption modeling to characterize the pharmacokinetic profiles of oral extended release methylphenidate products in adults. *PloS one* **2016,** *11* (10), e0164641.
